# Discrepancies in Demand of Internet of Things Services Among Older People and People With Disabilities, Their Caregivers, and Health Care Providers: Face-to-Face Survey Study

**DOI:** 10.2196/16614

**Published:** 2020-04-15

**Authors:** Heayon Lee, Yu Rang Park, Hae-Reong Kim, Na Young Kang, Gahee Oh, Il-Young Jang, Eunju Lee

**Affiliations:** 1 Division of Geriatrics, Department of Internal Medicine Asan Medical Center University of Ulsan College of Medicine Seoul Republic of Korea; 2 Department of Biomedical System Informatics Yonsei University College of Medicine Seoul Republic of Korea; 3 Hinda and Arthur Marcus Institute for Aging Research Hebrew SeniorLife Boston, MA United States

**Keywords:** Internet of Things, older adults, disability, health care, mobile phone

## Abstract

**Background:**

Home Internet of Things (IoT) services and devices have the potential to aid older adults and people with disabilities in their living environments. IoT services and devices can also aid caregivers and health care providers in conveniently providing care to those in need. However, real-world data on the IoT needs of vulnerable people are lacking.

**Objective:**

The objective of this study is to conduct a face-to-face survey on the demand for IoT services among older people and people with disabilities, their caregivers, and health care providers in a real-world setting and to see if there are any differences in the aspects of need.

**Methods:**

We conducted a face-to-face survey with 500 participants between January 2019 and March 2019. A total of 300 vulnerable people (200 older adults aged ≥65 years and 100 physically disabled people aged 30-64 years) were randomly sampled from either a population-based, prospective cohort study of aging—the Aging Study of Pyeongchang Rural Area (ASPRA)—or from the outpatient clinics at the Asan Medical Center, Seoul, South Korea. Simultaneously, their caregivers (n=150) and health care providers (n=50) participated in the survey. Detailed socioeconomic status, digital literacy, health and physical function, and home IoT service needs were determined. Among all commercially available IoT services, 27 services were classified into five categories: emergency and security, safety, health care, convenience (information), and convenience (operation). The weighted-ranking method was used to rank the IoT needs in different groups.

**Results:**

There were discrepancies in the demand of IoT services among the vulnerable groups, their caregivers, and health care providers. The home IoT service category that was required the most by the vulnerable groups and their caregivers was emergency and security. However, health care providers indicated that the safety category was most needed by the older adults and disabled people. Home IoT service requirements differed according to the different types of disabilities among the vulnerable groups. Participants with fewer disabilities were more willing to use IoT services than those with more disabilities.

**Conclusions:**

Our survey study shows that there were discrepancies in the demand of IoT services among the vulnerable groups, their caregivers, and health care providers. IoT service requirements differed according to the various types of disabilities. Home IoT technology should be established by combining patients’ priorities and individualized functional assessments among vulnerable people.

**Trial Registration:**

Clinical Research Information Service (CRIS; KCT0004157); https://tinyurl.com/r83eyva

## Introduction

The Internet of Things (IoT) is an emerging technology that connects a variety of everyday devices and systems, such as sensors, appliances, actuators, computers, and cellular phones, leading toward a highly distributed intelligent system capable of communicating with other devices and human beings [1]. Applications of IoT services and devices have the potential to aid older people and the physically disabled in their homes. IoT can help vulnerable groups to *age in place*, which is a concept whereby older people are able to continue living in their own homes as they age despite changes to their health and mobility [2,3]. In recent years, progress in wearable devices and sensor technologies have started to improve the prospects of services for assisting older people and physically disabled people [4-7]. It is agreed that helping the elderly and disabled people to live independently instead of in health care facilities provides cost savings and has significant potential to enhance quality of life [8-10]. Since some of the elderly and disabled people rely entirely on their family members for their assisted-care needs, their family caregivers may have emotional stress. Therefore, IoT can also aid their family caregivers and health care providers to conveniently give care and monitor those in need, as well as provide relief for family members [11-13].

Although a lot of work is going on regarding IoT-based care of older adults and disabled people, the adoption of these services is still quite low [14-16]. Among older adults and people with disabilities and their family members, older adults especially are generally slow to adopt emerging technologies, as they often have difficulties using electronic devices and they are concerned about their privacy [14]. Also, the concept of smart homes and IoT is relatively new; therefore, poor understanding about new innovative solutions can also be a factor of slow adoption, as well as cost [16-18].

Understanding the needs of vulnerable groups and their specific requirements is the key to success of the smart homes meant for their well-being. Yet, there is a lack of research on the needs of older adults and disabled people regarding smart homes and IoT [10]. The objective of this study was to investigate the demand for IoT services and devices among older people and disabled people, their caregivers, and health care providers in the real-world setting, and to see if there are any differences in the aspects of need according to the different types of disabilities among the groups.

## Methods

### Study Design and Recruitment of Study Population

This study was designed to investigate the needs for IoT in everyday life from the perspectives of older adults or people with disabilities. For this, we conducted a face-to-face survey with 500 participants between January 2019 and March 2019. We randomly selected 300 participants who were physically vulnerable (200 older adults aged ≥65 years and 100 disabled participants aged 30-64 years), 150 participants who were their caregivers, and 50 health care providers to participate in the survey.

We randomly selected 200 older adults with scores of 4-9 (ie, mild-to-moderate limitations in physical performance) on the Short Physical Performance Battery (SPPB) [19,20], with or without disability: 120 participants were from a population-based, prospective cohort study of aging—the Aging Study of Pyeongchang Rural Area (ASPRA)—and 80 participants were from the outpatient clinics at the Asan Medical Center, Seoul, South Korea. The ASPRA cohort was established in the Pyeongchang rural area, located 180 kilometers east of Seoul, South Korea, and has been described elsewhere [21,22].

In the disabled people’s group, we randomly selected 100 participants with (1) three or more comorbidities and/or (2) physical disabilities or mobility disability (ie, inability to walk more than 400 meters) [23]. Among them, 60 subjects were from the outpatient clinics at the Asan Medical Center and 40 participants were from the Pyeongchang rural area [24].

We also conducted a companion face-to-face survey on 150 caregivers, who were either trained professionals (n=5) or family members (n=145), and 50 health care providers, who were described as physicians, nurses, visiting nurses, and community care workers who regularly interact with older adults or people with disabilities. The caregivers and health care providers were asked to answer, from the perspectives of the vulnerable groups, what IoT services and devices they needed. All participants provided written informed consent. This study was approved by the Institutional Review Board of the Asan Medical Center (Institutional Review Board No. 2019-0041).

### Questionnaire Items

We developed paper questionnaires based on several publications [24-27] and conducted a face-to-face survey for about 30 minutes. In order to ensure the validity of the questionnaire, the questionnaire items were developed through several revisions with five experts. The questionnaire was composed of four parts and consisted of three versions, with respect to respondents: vulnerable groups (see Multimedia Appendix 1), caregivers, and health care providers. We researched all commercially available IoT services in the Korean market as of October 1, 2018. There were 84 services that were available. We removed IoT services and devices that had duplicate functions and grouped similar services, resulting in 27 services that were included in the questionnaire. We classified these services into five categories: security and emergency, safety, health care, convenience (information), and convenience (operation; see Table 1).

**Table 1:** Available Internet of Things (IoT) services by category.

Category	Services
Security	Home security and closed-circuit television (CCTV); Smart band and mobile *SOS* bell; Smart home *SOS* bell; Front door smart sensor; Voice-recognition front door lock
Safety	IoT-based power-system protection device; IoT-based smart gas monitoring; GPS trackers; Gas-valve remote control; Track use of smart water purifier
Health care	Smart home air purifier; Doctor’s appointments; Fitness program using smart television (TV); House temperature and humidity control; Smart IoT chair
Convenience (information)	Schedule appointments and alarms; Bus arrival time notification; Weather forecast; Traffic information
Convenience (operating)	Taxi call; Food order and delivery; Robot vacuum cleaner; Smart washing machine mobile app; Voice-recognition radio remote system; Voice-recognition TV remote system; Smart light switch; Voice-recognition alarm setting

Vulnerable groups were asked questions about digital literacy [28] (ie, using the internet or smartphones and their willingness to use these devices). Questions about perceptions on knowledge of IoT, actual use of IoT, and willingness to use IoT services were then asked. Lastly, participants were asked to choose which IoT services they needed the most on a scale of 1 (*the most needed*) to 10 (*the tenth-most needed*). Caregivers and health care providers were asked the same questions from the points of view of the vulnerable groups.

Questions about the following characteristics were asked: baseline socioeconomic status, age, and sex of vulnerable group members; caregiver status (ie, age, sex, number of visits per week, and average time of stay per visit); living area (ie, rural or urban); type of dwelling (ie, apartment, house, or semibasement house); monthly income (ie, <US $1000, US $1000-$2000, or >US $2000); literacy level (ie, proficient, basic-to-intermediate, or below basic); and television-watching behavior (ie, all the time vs as needed). To evaluate disability, we used validated scales for Koreans to assess dependence in six activities of daily living (ADL)—toileting, feeding, dressing, grooming, physical ambulation, and bathing—and in eight instrumental activities of daily living (IADL)—using the telephone, shopping, food preparation, housekeeping, laundry, using public transportation, taking personal medication, and the ability to handle finances [29]. ADL and IADL scores were rated on a scale of 0-100: 0 (*total dependence*), 25 (*extensive assistance*), 50 (*limited assistance*), 75 (*supervision only*), and 100 (*independent*). Disability was defined if assistance was required from another person in performing any of the above activities in ADL and/or IADL [29].

Information was obtained on comorbidities (ie, hypertension, hyperlipidemia, diabetes, cardiovascular disease, cerebrovascular disease, thyroid disease, biliary disease, osteoporosis, chronic liver disease, renal or urinary tract stone, asthma, tuberculosis, gastric or duodenal ulcer, gout, osteoarthritis, rheumatoid arthritis, depression, dementia, Parkinson disease, and cancer) and clinical symptoms (ie, fatigue, respiratory symptoms, constipation, poor oral intake, palpitation or chest discomfort, headache, dizziness, falls, weight loss, edema, depression, anxiety, insomnia or sleep disorders, memory loss, wandering, destructive behavior, and hallucinations). Levels in hearing (*bad, average,* or *good*), vision (*bad, average,* or *good*), and speech (*bad, average,* or *good*) were self-reported. Sensory disability was defined if the level of either hearing, vision, or speech was *bad*.

When comparing the IoT needs regarding underlying conditions and disability, we grouped the number of total ADL disabilities, number of total IADL disabilities, number of comorbidities, number of clinical symptoms, and number of triple (ie, hearing, visual, and speech) impairments into three groups: 0 (*no deficit*), 1-X (*mild-to-moderate deficit*), and >X (*severe deficit*), where the value of X is different for each impairment.

### Statistical Analysis

Analyses were conducted using R software, version 3.5.3 (The R Foundation), and Microsoft Excel, version 2016. Descriptive statistics for proportions of respondents, work profiles (eg, work experience, area of expertise, and institution), and responses regarding data demand, data linking, and deidentification were explored. The descriptive analysis examined differences in terms of five categories of IoT needs and deidentification processes. In the case of continuous data, we used the one-way analysis of variance (ANOVA) test to identify the differences in IoT needs between the groups. In the case of categorical data, we looked at the differences between the groups using the chi-square test. In general, when the expectation frequency was small, the Fisher exact test was used, except when there were more than three categories. The weighted-ranking method was used to rank the IoT needs in different groups. We asked for up to 10 responses per person for IoT needs. The higher the rank number, the lower the weight value, and vice versa: for example, the most important IoT needs have the lowest rank number (ie, 1), so are given the highest weight (ie, 10); on the other hand, the least important IoT needs have the highest rank number (ie, 10), so are given the lowest weight (ie, 1). Also, the weight was multiplied by the number of people who actually selected the services, and the value of the weighted sum was ranked.

## Results

### Socioeconomic Characteristics of Vulnerable Groups

The socioeconomic characteristics of the older adults and disabled people are seen in Table 2. The number of visits per week (mean 6.4, SD 1.6, vs mean 5.8, SD 2.2) and average hours per stay of the main caregivers (mean 19.7, SD 7.7, vs mean 17.8, SD 9.7) were significantly higher in the disabled people’s group compared to the older adults’ group. A total of 64.0% (128/200) of older adults lived in rural areas compared to 50.0% (50/100) of disabled people. The majority of people lived in houses in both groups. The majority (144/200, 72.0%) of older adults had monthly incomes of less than US $1000, whereas in the disabled people’s group, 35.0% (35/100) had monthly incomes of more than US $2000 and 25.0% (25/100) had monthly incomes of less than US $1000. Literacy levels were higher among the disabled people’s group compared to the older adults’ group (93/100, 93.0%, vs 131/200, 65.5%). Older adults tended to watch more television than disabled people. All of the results were statistically significant (*P*<.05).

**Table 2:** Socioeconomic characteristics of the vulnerable groups.

Characteristic		Older adults (n=200)	Disabled people (n=100)	*P* value
Total group members (n=300), n (%)		200 (66.7)	100 (33.3)	
**Caregiver, mean (SD)**				
	Number of visits per week	5.8 (2.2)	6.4 (1.6)	.008
	Average time of stay per visit (hours)	17.8 (9.7)	19.7 (7.7)	.07
Living area (rural), n (%)		128 (64.0)	50 (50.0)	.03
**Type of dwelling, n (%)**				**<.001**
	Apartment	39 (19.5)	35 (35.0)	
	House	157 (78.5)	52 (52.0)	
	Semibasement house	3 (1.5)	6 (6.0)	
**Monthly income (US $), n (%)**				**<.001**
	<1000	144 (72.0)	25 (25.0)	
	1000-2000	14 (7.0)	18 (18.0)	
	>2000	23 (11.5)	35 (35.0)	
**Literacy level, n (%)**				**<.001**
	Proficient	131 (65.5)	93 (93.0)	
	Basic-to-intermediate	34 (17.0)	4 (4.0)	
	Below basic	32 (16.0)	0 (0)	
**Television-watching behavior, n (%)**				**.002**
	All the time	95 (47.5)	26 (26.0)	
	As needed	86 (43.0)	57 (57.0)	

### Perception of Digital Literacy

The difference in perception of digital literacy based on the internet, smartphone use, and IoT is shown among the two vulnerable groups, their caregivers, and health care providers (see Table 3). The mean ages of older adults and disabled people were 78.13 years (SD 6.00) and 52.65 years (SD 10.19), respectively. A total of 33.5% (67/200) of older adults were male compared to 44.0% (44/100) of disabled people. Older adults tended to use the internet (22/200, 11.0%, vs 62/100, 62.0%) and smartphones (78/200, 39.0%, vs 88/100, 88.0%) less than did the disabled people. However, the proportion of older adults and disabled people willing to use the internet (81/200, 40.5%, vs 72/100, 72.0%) or smartphones (115/200, 57.5%, vs 62/100, 62.0%) was relatively high. Each of their caregivers replied similarly, showing matching trends.

In terms of IoT, the proportion of older adults that had heard of IoT was much lower compared to disabled people (25/200, 12.5%, vs 66/100, 66.0%). Only 1.0% (2/200) of older adults were currently using IoT services, whereas 20.0% (20/100) of disabled people were using them. The proportion of older adults willing to use IoT services in the future increased up to 57.5% (115/200) compared to 90.0% (90/100) of the disabled people. Each of their caregivers replied similarly, showing resembling trends.

The IoT needs in total and in five categories were presented as multiple-choice questions. The older adults indicated that security (125/384, 32.6%) was most needed, followed by safety (112/384, 29.2%) and then convenience (operation; 99/384, 25.8%). Their caregivers replied in the same order. The disabled group members indicated that security (76/254, 29.9%) was most needed, followed by convenience (operation; 61/254, 24.0%) and then safety (52/254, 20.5%). Their caregivers replied slightly differently. The health care providers also indicated that security (46/191, 24.1%) was most needed, followed by safety (45/191, 23.6%) and then convenience (operation; 37/191, 19.4%).

**Table 3:** Digital literacy and Internet of Things (IoT) needs of the three groups.

Category		Vulnerable group (n=300)				Caregivers (n=150)				Health care providers (n=50)		Total participants (N=500)	
		Older adults (n=200)		Disabled people (n=100)		Older adults (n=100)		Disabled people (n=50)					
Age (years), mean (SD)		78.13 (6.00)		52.65 (10.19)		65.39 (13.45)		51.40 (15.68)		38.88 (9.34)		63.89 (17.00)	
Gender (male), n (%)		67 (33.5)		44 (44.0)		51 (51.0)		16 (32)		5 (10)		183 (36.6)	
**ICT^a^ perception, n (%)**													
	Able to use internet		22 (11.0)		62 (62.0)		14 (14.0)		34 (68)		N/A^b^		132 (29.3)^c^
	Willing to use internet		81 (40.5)		72 (72.0)		46 (46.0)		37 (74)		N/A		236 (52.4)^c^
	Able to use smartphone		78 (39.0)		88 (88.0)		42 (42.0)		44 (88)		N/A		252 (56.0)^c^
	Willing to use smartphone		115 (57.5)		67 (67.0)		62 (62.0)		43 (86)		N/A		287 (63.8)^c^
	Heard of IoT		25 (12.5)		66 (66.0)		7 (7.0)		26 (52)		N/A		124 (27.6)^c^
	Currently using IoT		2 (1.0)		20 (20.0)		4 (4.0)		13 (26)		N/A		39 (8.7)^c^
	Willing to use IoT		115 (57.5)		90 (90.0)		63 (63.0)		45 (90)		N/A		313 (69.6)^c^
**IoT needs by service category, n (%)**													
	Total^d^		384 (100)		254 (100)		307 (100)		166 (100)		191 (100)		1302 (100)
	Security		125 (32.6)		76 (29.9)		82 (26.7)		45 (27.1)		46 (24.1)		374 (28.73)
	Safety		112 (29.2)		52 (20.5)		81 (26.4)		41 (24.7)		45 (23.6)		331 (25.42)
	Health care		26 (6.8)		44 (17.3)		42 (13.7)		29 (17.5)		31 (16.2)		172 (13.21)
	Convenience (information)		22 (5.7)		21 (8.3)		26 (8.5)		17 (10.2)		32 (16.8)		118 (9.06)
	Convenience (operation)		99 (25.8)		61 (24.0)		76 (24.8)		34 (20.5)		37 (19.4)		307 (23.58)

^a^ICT: information and communication technology.

^b^N/A: not applicable.

^c^Total number of respondents was 450.

^d^Respondents could select multiple needs; individual category percentages are based on the total IoT needs in each column.

### Internet of Things Needs by Category Depending on Underlying Conditions and Disability Types in Vulnerable Groups

Tables 4 and 5 show the difference in underlying characteristics and conditions in terms of IoT category needs in vulnerable groups. In older adults, people with lower incomes paid more attention to security and safety, whereas people with higher incomes paid more attention to health care or convenience (information), and these findings were statistically significant (see Table 4). Among the people who were physically disabled, those with lower incomes paid more attention to safety and convenience (operating), whereas people with higher incomes paid more attention to health care, however, these results were not significant (see Table 5). In older adults, variations in smartphone use, ADL, and IADL caused significant differences in IoT service category needs. However, among physically disabled people, there were no significant differences.

**Figure 1:**
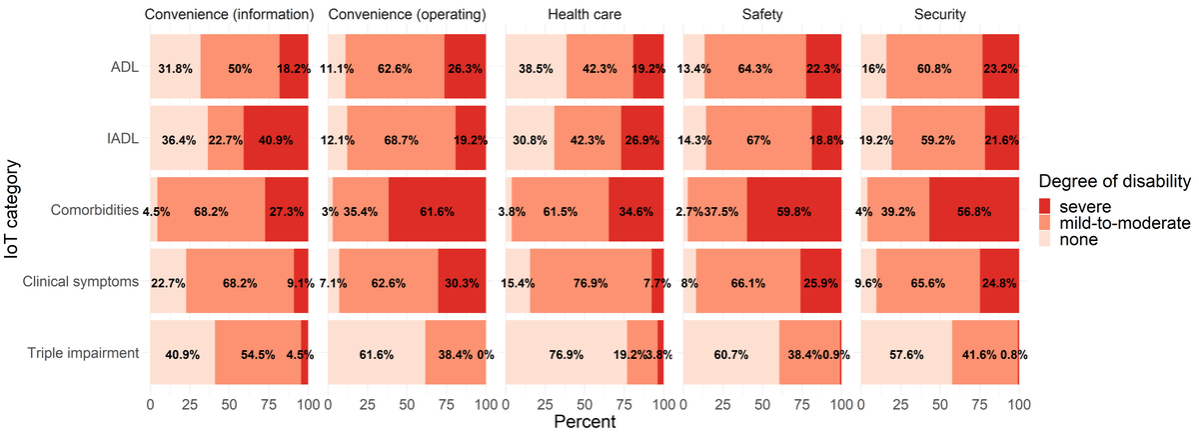
The total number of Internet of Things (IoT) needs by category, as a function of underlying conditions in older adults. ADL: activities of daily living; IADL: instrumental activities of daily living; Triple impairment: hearing, vision, and speech impairments.

**Figure 2:**
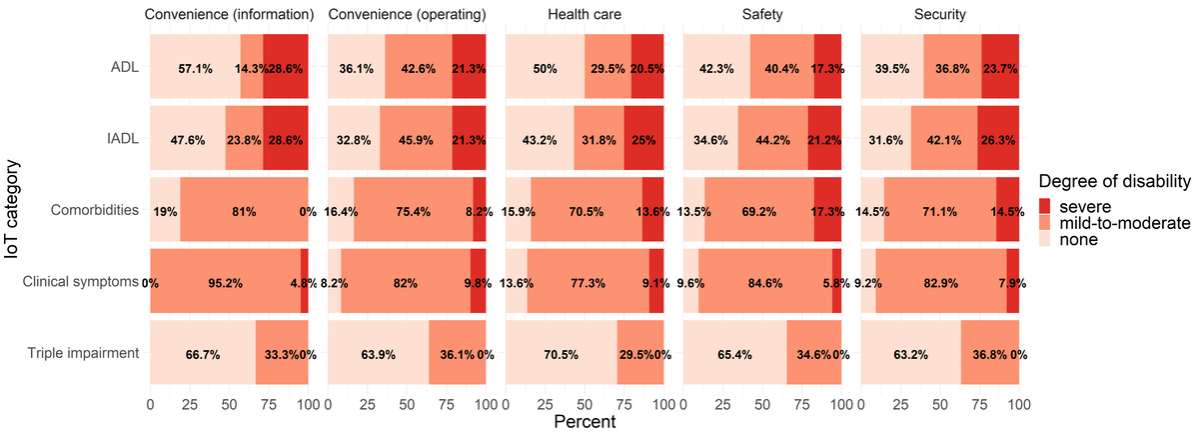
The total number of Internet of Things (IoT) needs by category, as a function of underlying conditions in disabled people. ADL: activities of daily living; IADL: instrumental activities of daily living; Triple impairment: hearing, vision, and speech impairments.

Figures 1 and 2 show IoT needs—multiple replies were possible—depending on disability type among both vulnerable groups. Among both older adults (see Figure 1) and disabled people (see Figure 2), those with mild-to-moderate disabilities were more willing to use IoT services than those with severe disabilities. People with no sensory disabilities (ie, triple impairment in hearing, vision, and speech) were more willing to use IoT services than people with mild-to-moderate sensory disabilities in both groups.

**Table 4:** Differences in underlying characteristics and conditions in terms of Internet of Things (IoT) category needs in older adults.

Variables			IoT needs by service category (n=384 total selections), n (%)					*P* value	
			Security (n=125)	Safety (n=112)	Health care (n=26)	Convenience (information) (n=22)	Convenience (operating) (n=99)		
Age (years), mean (SD)			78.26 (5.76)	78.33 (5.78)	73.54 (5.36)	76.59 (6.77)	78.88 (5.75)		.001
Gender (male)			41 (32.8)	34 (30.4)	15 (58)	13 (59)	31 (31)		.009
**Living area**									**<.001**
	Rural	81 (64.8)		82 (73.2)	6 (23)	1 (4)	72 (73)		
	Urban	44 (35.2)		30 (26.8)	20 (77)	21 (95)	27 (27)		
**Type of dwelling**									**<.001**
	Apartment	22 (17.6)		17 (15.2)	11 (42)	12 (54)	15 (15)		
	House	103 (82.4)		95 (84.8)	15 (58)	10 (45)	84 (85)		
**Monthly income (US $)**									**<.001**
	<1000	102 (81.6)		94 (83.9)	15 (58)	11 (50)	87 (88)		
	1000-2000	8 (6.4)		5 (4.5)	3 (11)	6 (27)	5 (5)		
	>2000	15 (12.0)		13 (11.6)	8 (31)	5 (23)	7 (7)		
**Literacy level**									**.28**
	Below basic	23 (18.4)		23 (20.5)	2 (8)	1 (4)	18 (18)		
	Basic-to-intermediate	22 (17.6)		23 (20.5)	2 (8)	2 (9)	18 (18)		
	Proficient	80 (64.0)		66 (58.9)	22 (85)	19 (86)	63 (64)		
**Television-watching behavior**									**.07**
	As needed	53 (42.4)		42 (37.5)	14 (54)	12 (54)	38 (38)		
	Do not watch	12 (9.6)		8 (7.1)	4 (15)	5 (23)	8 (8)		
	All the time	60 (48.0)		62 (55.4)	8 (31)	5 (23)	53 (53)		
**ICT^a^ perception**									
	Able to use internet	12 (9.6)		8 (7.1)	4 (15)	4 (18)	6 (6)		.23
	Willing to use internet	56 (44.8)		52 (46.4)	17 (65)	12 (54)	43 (43)		.27
	Able to use smartphone	50 (40.0)		40 (35.7)	22 (85)	11 (50)	34 (34)		<.001
	Willing to use smartphone	75 (60.0)		69 (61.6)	15 (58)	8 (36)	58 (59)		.29
	Able to use IoT	1 (0.8)		1 (0.9)	0 (0)	0 (0)	1 (1)		.98
	Willing to use IoT	73 (58.4)		75 (67.0)	16 (61)	10 (45)	61 (62)		.37
**ADL^b^ deficits**									**.04**
	None (0)	20 (16.0)		15 (13.4)	10 (38)	7 (32)	11 (11)		
	Mild-to-moderate (1-3)	76 (60.8)		72 (64.3)	11 (42)	11 (50)	62 (63)		
	Severe (4-6)	29 (23.2)		25 (22.3)	5 (19)	4 (18)	26 (26)		
**IADL^c^ deficits**									**.004**
	None (0)	24 (19.2)		16 (14.3)	8 (31)	8 (36)	12 (12)		
	Mild-to-moderate (1-4)	74 (59.2)		75 (67.0)	11 (42)	5 (23)	68 (69)		
	Severe (5-8)	27 (21.6)		21 (18.8)	7 (27)	9 (41)	19 (19)		
**Clinical symptoms**									**.11**
	None (0)	12 (9.6)		9 (8.0)	4 (15)	5 (23)	7 (7)		
	Mild-to-moderate (1-9)	82 (65.6)		74 (66.1)	20 (77)	15 (68)	62 (63)		
	Severe (>10)	31 (24.8)		29 (25.9)	2 (8)	2 (9)	30 (30)		
**Comorbidity**									**.07**
	None (0)	5 (4.0)		3 (2.7)	1 (4)	1 (4)	3 (3)		
	Mild-to-moderate (1-3)	49 (39.2)		42 (37.5)	16 (61)	15 (68)	35 (35)		
	Severe (4-6)	71 (56.8)		67 (59.8)	9 (35)	6 (27)	61 (62)		
**Triple impairment^d^**									**.13**
	None (0)	72 (57.6)		68 (60.7)	20 (77)	9 (41)	61 (62)		
	Mild-to-moderate (1-2)	52 (41.6)		43 (38.4)	5 (19)	12 (54)	38 (38)		
	Severe (3)	1 (0.8)		1 (0.9)	1 (4)	1 (4)	0 (0)		

^a^ICT: information communication and technology.

^b^ADL: activities of daily living.

^c^IADL: instrumental activities of daily living.

^d^Hearing, visual, and speech impairments.

**Table 5:** Differences in underlying characteristics and conditions in terms of Internet of Things (IoT) category needs in disabled people.

Variables		IoT needs by service category (n=254 total selections), n (%)					*P* value	
		Security (n=76)	Safety (n=52)	Health care (n=44)	Convenience (information) (n=21)	Convenience (operating) (n=61)		
Age (years), mean (SD)		53.11 (10.10)	52.90 (10.37)	51.77 (9.81)	53.52 (9.02)	51.75 (10.85)		.89
Gender (male)		34 (45)	23 (44)	10 (23)	7 (33)	28 (46)		.10
**Living area**								**.26**
	Rural	42 (55)	33 (63)	20 (45)	8 (38)	33 (54)		
	Urban	34 (45)	19 (36)	24 (54)	13 (62)	28 (46)		
**Type of dwelling**								**<.001**
	Apartment	28 (37)	19 (36)	22 (50)	12 (57)	23 (38)		
	House	46 (60)	33 (63)	21 (48)	8 (38)	37 (61)		
**Income (US $)**								**.63**
	<1000	33 (43)	26 (50)	14 (32)	8 (38)	29 (47)		
	1000-2000	15 (20)	11 (21)	9 (20)	6 (29)	14 (23)		
	>2000	28 (37)	15 (29)	21 (48)	7 (33)	18 (29)		
**Literacy level**								**.98**
	Below basic	1 (1)	1 (2)	1 (2)	0 (0)	1 (2)		
	Basic-to-intermediate	4 (5)	4 (8)	2 (4)	0 (0)	4 (7)		
	Proficient	71 (93)	47 (90)	41 (93)	21 (100)	56 (92)		
**Television-watching behavior**								**.56**
	As needed	43 (57)	32 (61)	31 (70)	15 (71)	34 (56)		
	Do not watch	12 (16)	5 (10)	7 (16)	3 (14)	11 (18)		
	All the time	21 (28)	15 (29)	6 (14)	3 (14)	16 (26)		
**ICT^a^ perception**								
	Able to use internet	54 (71)	36 (69)	30 (68)	16 (76)	43 (70)		.97
	Willing to use internet	56 (74)	40 (77)	28 (64)	14 (67)	43 (70)		.80
	Able to use smartphone	67 (88)	43 (83)	40 (91)	20 (95)	52 (85)		.56
	Willing to use smartphone	52 (68)	39 (75)	27 (61)	13 (62)	42 (69)		.85
	Able to use IoT	12 (16)	7 (13)	8 (18)	4 (19)	14 (23)		.76
	Willing to use IoT	68 (89)	46 (88)	41 (93)	20 (95)	57 (93)		.82
**ADL^b^ deficits**								**.45**
	None (0)	30 (39)	22 (42)	22 (50)	12 (57)	22 (36)		
	Mild-to-moderate (1-3)	28 (37)	21 (40)	13 (29)	3 (14)	26 (43)		
	Severe (4-6)	18 (24)	9 (17)	9 (20)	6 (29)	13 (21)		
**IADL^c^ deficits**								**.68**
	None (0)	24 (32)	18 (35)	19 (43)	10 (48)	20 (33)		
	Mild-to-moderate (1-4)	32 (42)	23 (44)	14 (32)	5 (24)	28 (46)		
	Severe (5-8)	20 (26)	11 (21)	11 (25)	6 (29)	13 (21)		
**Clinical symptoms**								**.81**
	None (0)	7 (9)	5 (10)	6 (14)	0 (0)	5 (8)		
	Mild-to-moderate (1-9)	63 (83)	44 (85)	34 (77)	20 (95)	50 (82)		
	Severe (>10)	6 (8)	3 (6)	4 (9)	1 (5)	6 (10)		
**Comorbidity**								**.68**
	None (0)	11 (14)	7 (13)	7 (16)	4 (19)	10 (16)		
	Mild-to-moderate (1-3)	54 (71)	36 (69)	31 (70)	17 (81)	46 (75)		
	Severe (4-6)	11 (14)	9 (17)	6 (14)	0 (0)	5 (8)		
**Triple impairment^d^**								**.95**
	None (0)	48 (63)	34 (65)	31 (70)	14 (67)	39 (64)		
	Mild-to-moderate (1-2)	28 (37)	18 (35)	13 (29)	7 (33)	22 (36)		
	Severe (3)	0 (0)	0 (0)	0 (0)	0 (0)	0 (0)		

^a^ICT: information communication and technology.

^b^ADL: activities of daily living.

^c^IADL: instrumental activities of daily living.

^d^Hearing, visual, and speech impairments.

### Weighted Rankings of Internet of Things Needs Depending on Different Groups

**Figure 3:**
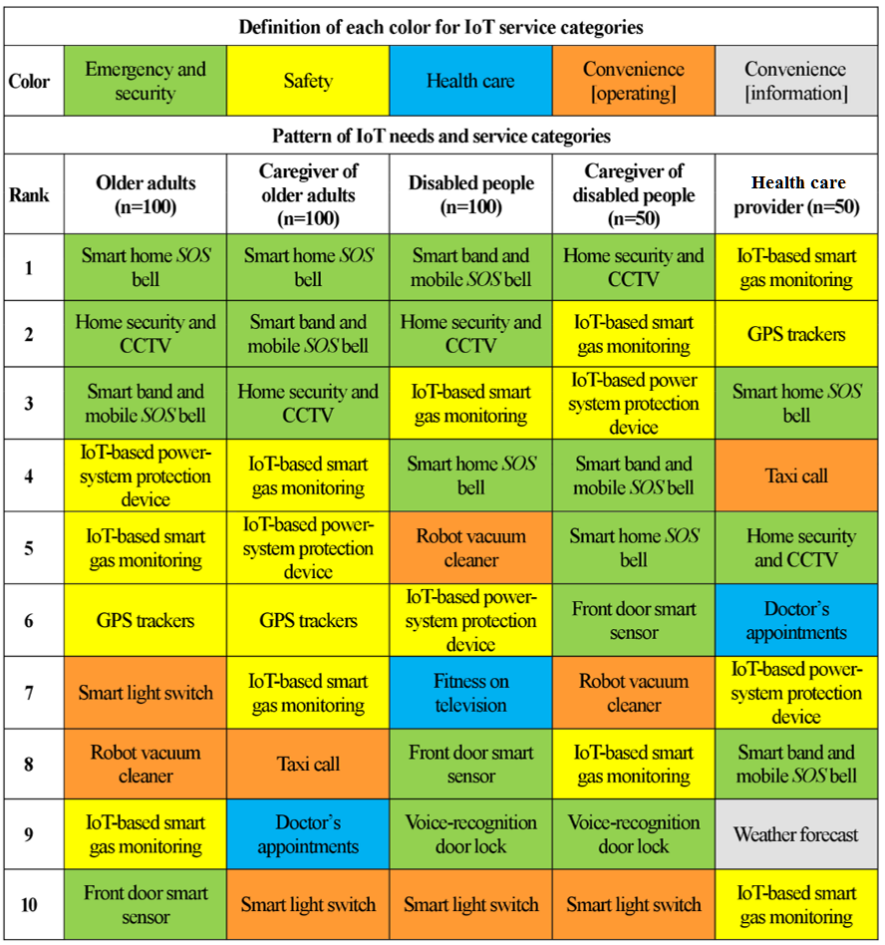
Weighted rankings of Internet of Things (IoT) services and needs depending on different groups. CCTV: closed-circuit television.

Figure 3 shows weighted rankings of IoT needs depending on various groups. The top-three IoT services (ie, ranked 1-3) for older adults were *smart home SOS bell*, *home security and closed-circuit television (CCTV)*, and *smart band and mobile SOS bell services*; these were within the emergency and security category. Although the order of the service rankings were slightly different, their caregivers replied similarly in both groups. The services ranked 4-6 were also similar in both groups. The services they selected were all in the safety categories: *IoT-based power-system protection device*, *IoT-based smart gas monitoring*, and *GPS tracker service*.

The top-two IoT services (ie, ranked 1 and 2), for disabled people were *smart band and mobile SOS bell* and *home security and CCTV*, which were both in the emergency and security category. The service ranked as number 3 was *IoT-based smart gas monitoring*, which was in the safety category. Their caregivers replied slightly differently. The *home security and CCTV* service topped the ranks in the emergency and security category; the *IoT-based smart gas monitoring* service was ranked as number 2 and *IoT-based power-system protection device* was ranked as number 3, both of which were in the safety category.

In the health care provider group, participants’ top-two IoT services (ie, ranked 1 and 2) were *smart gas monitoring* and *GPS tracker*, both in the safety category, which was different from the other groups. The service ranked as number 3 was *smart home SOS bell* in the emergency and security category.

## Discussion

### Principal Findings

The purpose of this study was to conduct a face-to-face survey on the demand for IoT among older people and people with disabilities, their caregivers, and health care providers in the real-world setting and to see if there are any differences in the aspects of need. The primary finding of this study was that IoT service needs were different among the vulnerable groups, their caregivers, and health care providers. The most required IoT service category selected by the vulnerable groups and their caregivers was security. Meanwhile, health care providers decided that IoT services in the safety category were most needed by the vulnerable groups. In addition, IoT service preferences differed according to various types of disabilities in the vulnerable groups.

Smart home technology has been anticipated to be at the front line of individualized health care, yet there are still hurdles that prevent home IoT technology from spreading among vulnerable populations. Various studies have addressed learning and adherence issues as the main problems [30-32]. Even with well-targeted populations, wearable-device studies have reported only 10% use within one year of incentive shrinkage [31]. Other studies also showed that the adoption rates of IoT services and devices are still very low and were reported to be around 5%-15% in older adults and physically disabled people [30]. However, previous studies have been focused on the technical aspects or digital literacy only in determining the causes of low adoption. In our study, we showed that the percentage of IoT use in older adults and physically disabled people was still very low regardless of underlying literacy or digital literacy levels. The reason for this was mainly due to lack of knowledge and exposure to the IoT devices compared with internet or smartphone exposure, especially in older adults. Therefore, education on using and experiencing these devices is needed to improve adoption, as our results showed that there is an interest and willingness to use IoT devices among the vulnerable groups.

One of our study’s strengths is that we explored the different perspectives of IoT demands that have been subject to stereotypes and insufficiently studied in real-world settings [17,33]. We showed the actual needed IoT service categories and rankings that the users, their caregivers, and health care providers chose in person. Vulnerable people are very different physically, mentally, and environmentally from the general population; therefore, patient-centered approaches should be emphasized as they may improve outcomes for people with multiple chronic conditions [34]. We showed the users’ priorities based on their socioeconomic status, literacy levels, digital literacy levels, underlying disabilities, and remnant physical performances, which provide a comprehensive view for practical implementation of IoTs in older adults and disabled people.

Furthermore, our study results showed the IoT service needs from the perspective of the caregivers and health care providers, which is also important. Older adults and physically disabled people often rely on caregivers and health care providers for validation of behaviors, including purchase and use of technology [35]. Also, IoT-based systems can share information with them so they can intervene in case of emergency and provide support [36]. Our results were different in that the vulnerable groups were willing to try new technology, quite contrary to the social perception. We also showed that the IoT service needs of the caregivers who were mainly family were in agreement with the vulnerable group members, which proves that the caregivers were sensitive to the specific needs of their care recipients. Our results may have differed if most of the caregivers had been paid. Paid caregivers may have a different financial perspective than family members and, therefore, may have replied differently. Interestingly, the health care providers replied differently, which shows that their demands deviate from the needs of the vulnerable people and their families. We need to be aware of these discrepancies when recommending and applying IoT devices to vulnerable users.

### Internet of Things Service Categories in Each Group

The most required IoT services chosen by the vulnerable groups and their caregivers in our study were within the security category. It has been reported that vulnerable groups value independence, privacy, and social interactions, while they have negative impressions about personal emergency alarms because they are obtrusive and even shameful and they dislike being watched [37-40]. Our study showed otherwise. There was a strong need for security among vulnerable people and their family caregivers. Vulnerable people are physically less mobile and their activities mostly take place within the home environment. They can fear loneliness and isolation; however, they wish to remain independent as long as possible [41]. Therefore, the security services they selected, especially *SOS* alarms and wearables, can act as a backup plan for self-management and can be used in emergency situations to notify family members or caregivers and providers [42-45].

The health care providers were focused more on the safety category. The reason the vulnerable groups did not choose this service as their top priority in our study could be that they are still relatively independent and so it did not meet their demands [38,39]. This shows that health care providers did not fully comprehend the needs of the vulnerable groups and may have stereotyped them as all needing support in their daily activities. Health care providers should be aware of these discrepancies and, therefore, consider a patient-centered approach when considering IoT services and devices.

In all of the groups, services related to health care were the least popular. Previous IoT solutions in these vulnerable groups had been mainly designed for health monitoring, such as monitoring medical parameters, activity level, medical compliance, nutrition, fitness, and sleep [36,46-48]. However, health care–related services in our list were not favored by the users. Unfortunately, due to the Personal Information Protection Act and the Medical Service Law enacted in 2011 in South Korea, we are unable to use health care devices for remote monitoring regarding medical information security. Therefore, we could only select commercially available IoT devices that were related to health care and somewhat less common. Our results may have differed if the devices had been directly monitoring health. IoT regulation depends on the country and different domestic circumstances need to be acknowledged.

### Internet of Things Needs Vary Depending on Different Types of Disabilities

In our study, IoT needs varied due to the different combinations of disabilities. People with mild-to-moderate disabilities tended to respond more to needing IoT services compared to people with either no disabilities or severe disabilities. This indicates that, perhaps for people with severe disabilities, IoT services were too difficult to use or participants were too frail and needed continued care [49]. On the other hand, it is possible that people with no disabilities did not necessarily need the IoT services. Many of the IoT services designed to be used do not usually consider that the functional limitations of each user are different [42]. Therefore, the key challenge is IoT customization for older adults and people with disabilities. Individualized, comprehensive, functional assessment among vulnerable people to analyze their underlying conditions, functional status, and disabilities are recommended. We are currently undergoing trials to apply IoT devices for use among vulnerable people based on these assessments.

### Limitations

The main limitation of this study is that the respondents were mostly people with mild functional disabilities. However, the purpose of IoT services is to enhance usability among vulnerable people with mild functional disabilities before their conditions deteriorate. Although our study may not directly represent the opinions of the aging or disabled population, it does illuminate, through a cross-sectional approach, the present status of home IoT needs from the perspectives of users and their families in the real world. Further investigation into groups of people with severe functional disabilities is needed to represent the overall opinions of the population.

### Conclusions

Our survey study shows that there were inconsistencies in the demand of IoT services among vulnerable groups, their caregivers, and health care providers. IoT service requirements differed according to the various types of disabilities. Home IoT technology should be established by combining patients’ priorities and individualized functional assessments of vulnerable people in an environment where patient-centered approaches and collaborative decision making are emphasized. This information and future trial data can inform public health professionals and industry workers in designing home IoT services for vulnerable populations.

## Appendix

Multimedia Appendix 1 Questionnaire for older adults and physically disabled people.

## Abbreviations

ADL: activities of daily living
ANOVA: analysis of variance
ASPRA: Aging Study of Pyeongchang Rural Area
CCTV: closed-circuit television
IADL: instrumental activities of daily living
IoT: Internet of Things
KHIDI: Korea Health Industry Institute
SPPB: Short Physical Performance Battery
