# Supplementary material for: Discrepancies in Demand of Internet of Things Services Among Older People and People With Disabilities, Their Caregivers, and Health Care Providers: Face-to-Face Survey Study
Source: J Med Internet Res. 2020 Apr 15;22(4):e16614. doi: 10.2196/16614 (PMC7191341; doi:10.2196/16614)
Supplement: Multimedia Appendix 1 [file jmir_v22i4e16614_app1.docx]

Questionnaire: A) Older adults/Physically disabled version

**1. [Socioeconomic Status]**

| 1. What is your age? _____________  2. What is your sex? 1) Male 2) Female  3. What type of community do you currently live in? 1) Rural 2) Urban  4. What type of house do you live in?  1) Apartment 2) House 3) Semi-basement house  5. Who do you live with?  1) alone 2) spouse 3) children 4) neighbors 5) friend 6) paid caregiver  7) others _____________  6. Who is your usual caregiver?  1) none 2) spouse 3) children 4) neighbors 5) friend 6) paid caregiver 7) others ___________  7. What is the age of your caregiver? ____________  8. How much time do you spend with your caregiver?  1) ( ) times per week  2) Average ( ) hours per visit  9. What is your monthly income?  1) None 2) <300$ 3) 300-499$ 4) 500-999$  5) 1000-1499$ 6) 1500-1999$ 7) 2000-2999$ 8) > 3000$  10. What is your literacy level?  1) below basic 2) basic-intermediate 3) proficient  11. What is your level of hearing?  1) bad 2) average 3) good  12. What is your level of vision?  1) bad 2) average 3) good  13. What is the level of your speech?  1) bad 2) average 3) good |
| --- |

**2. [Digital Literacy]**

| 1. How many hours of TV do you watch per day?  1) none 2) as needed 3) all the time  2. Are you able to use the internet?  1) Yes 2) No  3. Are you willing to use the internet, if you have not in the past?  1) Yes 2) No  4. Are you able to use smart phones?  1) Yes 2) No  5. If you do not use smart phones what is the main reason?  1) No problem, can use it very well  2) Do not feel it is necessary  3) Complicated to use  4) Afraid to use  5) Expensive to buy  6) Expensive to pay monthly bill  6. Are you willing to use smart phones if you have not in the past?  1) Yes 2) No  7. Have you heard of IoTs (ie. Smartband, smart home, smart shoes)?  1) Never heard of it  2) Have heard of it, but not familiar  3) Am familiar to it  8. Are you able to use IoTs?  1) Yes 2) No  9. What is the reason for not using IoTs?  1) I have no problems using it  2) I do not know what it is and am afraid to use it  3) I do not feel it is necessary  4) I feel it is complicated to use  6) I feel it is too expensive to buy  7) I want to use it but have no smartphone  10. Are you willing to use IoTs, if you have not in the past?  1) Yes 2) No |
| --- |

**3. [IoT Service Need]**

| 1. Which IoT services are you currently using?  **A. Emergency & security**  **1) Home security and CCTV:** activity level monitoring, keeps your home safe from intruders and environmental threats like fires and floods  **2) Smart band/mobile SOS bell:** activity level monitoring, SOS bell call connected to families/caregivers or social services  **3) Smart home SOS bell:** usually in the living room or bathroom, activity level monitoring for detection of fall or other medical emergencies  **4) Front door smart sensor:** check access history to see who entered your home and at what time via an app, which records this information whenever your family unlocks the door  **5) Voice-recognition front door lock:** remote control is possible with voice recognition (ie. Alexa, google assistant, or Siri voice commands)  **B. Safety**  **6) IoT-based Power-system protection device:** real-time monitoring and notification of events, fast disconnection of the power supply in case of fault events like leakage current, overcurrent or overvoltage  **7) IoT-based smart gas monitoring:** iot detects gas leak and send the data to android app which controls gas leakage by closing the valve automatically  **8) GPS trackers:** wearable, share location updates to family via smart phone application  **9) Gas valve remote control:** users can remotely lock the gas valve, set temperature alerts, set timers and control gas valves in the homes of others  **10) Track use of smart water purifier:** water purifier use is tracked and if not used for a while then notification alarm goes to family to see if there have been emergencies such as fall or medical emergencies in the house  **C. Healthcare**  **11) Smart home air purifier:** connected to app to check on live air quality both indoors and  outdoors, remote control is available and can automatically adjust the airflow if sensors detect  a change in air conditions  **12) Doctors appointment:** by interfacing with hospital systems, smart healthcare  Solutions enable for appointment scheduling on mobile devices  **13) Fitness program using smart TV:** IoT linked programs on smart TV to monitor physical  exercise and activity, alarms to encourage exercise and right posture  **14) House temperature/humidity control:** connected to smartphone app to check both indoor  and outdoor, remote control available and can automatically adjust if sensors detect change  **15) Smart IoT chair:** makes users sit correctly with recognition of their own current state by  providing intuitive and visualized data real time to IoT chair sensor alarms  **D. Convenience (information)**  **16) Schedule appointment/alarm:** smart speaker that hears you from across the room, informs  you of your daily schedules and alarms  **17) Bus arrival time notification:** smart speaker that informs you of the bus arrival information  **18) Weather forecast:** smart speaker that informs you of the weather and current air pollution  state  **19) Traffic information:** smart speaker that informs you of the traffic information  **E. Convenience (operating)**  **20) Taxi call:** smart speaker that connects to smart phone taxi calling application  **21) Food order and delivery:** smart speaker that connects to smart phone food delivery  application  **22) Robot vaccum cleaner:** use a booking system in smart phone application to schedule the  automated services of a robot vaccum cleaner  **23) Smart washing machine mobile app:** smart washer application allows to control your  washer remotely from your smart phone and notifies the status of the washer  **24) Voice-recognition radio remote system:** smart speaker that recognizes your voice  command and turns on/off the radio  **25) Voice-recognition TV remote system:** smart speaker that recognizes your voice command  and turns on/off the TV  **26) Smart light switch:** smart speaker that recognizes your voice command and turns on/off  the light  **27) Voice-recognition alarm setting:** smart speaker that recognizes your voice command and  turns on/off the alarm  2. (Regardless of current use) Choose and place the IoT services in the above list in order of need from 1 – 10 (where 1 is the highest possible rank).  **<Rank order>**  1st ___________________________  2nd ___________________________  3rd ___________________________  4th ___________________________  5th ___________________________  6th ___________________________  7th ___________________________  8th ___________________________  9th ___________________________  10th __________________________ |
| --- |

**4. [Health status and activities of daily living]**

| 1. Do you have any underlying diseases? (multiple choice)  1) Hypertension 2) Hyperlipidemia) 3) Diabetes 4) Angina/MI 5) CVA/stroke 6) Thyroid ds  7) Biliary ds 8) Osteoporosis 9) Chronic hepatitis/liver cirrhosis 10) Renal stone/urinary stone  11) Asthma/allergy 12) Tuberculosis 13) Gastric/duodenal ulcer 14) Gout 15) Osteoarthritis  16) Rheumatoid arthritis 17) Depression 18) Dementia 19) Parkinson’s disease 20) Cancer  21) etc ____________________________  2. Do you have any following symptoms in the previous 1 month? (multiple choice)  1) fatigue  2) cough/ dyspnea  3) constipation  4) poor oral intake  5) palpitation/ chest discomfort  6) headache  7) dizziness  8) fall  9) edema  10) weight loss  3. Do you have any following mood symptoms in the previous 1 month? (multiple choice)  1) depression  2) anxiety  3) insomnia/ sleep disorders  4) memory loss  5) wandering  6) destructive behavior  7) hallucination  4. How many prescribed medications do you take daily?  1) none 2) 1~2 3) 3~4 4) more than 5~6 5) I don’t know  **5. Tasks related to personal care: Activities of Daily Living (ADL)**  **In each category, circle the scale that most closely describes your highest level of functioning.**   \| 0: Total dependence, 25: Extensive assistance, 50: Limited assistance,  75: Supervision only, 100: Independent \| \| --- \|     A. Toilet  0) 25) 50) 75) 100)  B. Feeding  0) 25) 50) 75) 100)  C. Dressing  0) 25) 50) 75) 100)  D. Grooming (neatness, hair, nails, hands, face, clothing)  0) 25) 50) 75) 100)  E. Physical Ambulation  0) 25) 50) 75) 100)  F. Bathing  0) 25) 50) 75) 100)  **6. Instrumental Activities of Daily Living (IADL)**  **In each category, circle the scale that most closely describes your highest level of functioning.**   \| 0: Total dependence, 25: Extensive assistance, 50: Limited assistance,  75: Supervision only, 100: Independent \| \| --- \|   A. Ability to use telephone  0) 25) 50) 75) 100)  B. Shopping  0) 25) 50) 75) 100)  C. Food preparation  0) 25) 50) 75) 100)  D. Housekeeping  0) 25) 50) 75) 100)  E. Laundry  0) 25) 50) 75) 100)  F. Using public transportation  0) 25) 50) 75) 100)  G. Taking personal medications  0) 25) 50) 75) 100)  H. Ability to handle finances  0) 25) 50) 75) 100) |
| --- | --- | --- |
